# Perceived barriers to the practice of preventive measures for COVID-19 pandemic among health professionals in public health facilities of the Gamo zone, southern Ethiopia: a phenomenological study

**DOI:** 10.1186/s12889-021-10256-3

**Published:** 2021-01-22

**Authors:** Abera Mersha, Shitaye Shibiru, Meseret Girma, Gistane Ayele, Agegnehu Bante, Mekidim Kassa, Sintayehu Abebe, Misgun Shewangizaw

**Affiliations:** 1 School of Nursing, College of Medicine and Health Sciences, Arba Minch University, Arba Minch, Ethiopia; 2 School of Public Health, College of Medicine and Health Sciences, Arba Minch University, Arba Minch, Ethiopia

**Keywords:** Coronavirus, Pandemic, Barriers, Phenomenological study, And Ethiopia

## Abstract

**Background:**

Novel coronavirus is a global pandemic and killed many individuals, including health care professionals. It caused stress on the health care system of all countries. Presently, studies are emerging regarding the COVID-19 pandemic in different aspects. However, a few have explored barriers that affecting the practice of preventive measures for the COVID-19. As such, the study aimed to fill these research gaps in the study setting.

**Methods:**

A semi-structured interview guide was used to conduct this phenomenological study among 16 key informants. Key informants were recruited by the purposive sampling method. To analyze that data, thematic content analysis was employed by using an inductive approach in NVivo 12 Pro software.

**Results:**

In this study, six main themes were identified with the sub-themes. Overview of COVID-19 pandemic (with the six sub-themes), consequences (with the two sub-themes), perceived practice (with four sub-themes), perceived barriers (with four sub-themes), newfangled activities (with three sub-themes), and suggestion for improvement (with seven sub-themes) were the major themes. The participants perceived the influence of shortage of personal protective equipment and solutions for hand hygiene, negligence and ignorance, inadequate infrastructure, lack of training, and lack of attention and recognition for the staff on the practice of preventive measures.

**Conclusions:**

This study showed a gap in preventive measure practices for the COVID-19 in the health care system. Community influences, health care provider related barriers, institutional barriers, and lack of communication and support affect the practice. Hence, attention should give to fulfill the necessary supplies in the health facilities, improve the infrastructures, and equip health professionals by providing capacity-building activities. Besides, health care workers must recognize, and attention is needed.

**Supplementary Information:**

The online version contains supplementary material available at 10.1186/s12889-021-10256-3.

## Background

Coronavirus disease-2019 (COVID-19) is a highly contagious acute respiratory disease. The causative agent is a novel coronavirus (2019-nCoV) and first evident in Wuhan City, Hubei province, China [1–6]. The known mode of transmission are droplets, contact, and aerosol [7, 8]. The complete clinical manifestation is not clear yet, but the most commonly reported symptoms are fever, cough, myalgia or fatigue, pneumonia, and complicated dyspnea [9–11]. Real-time fluorescence (RT-PCR) detects the nucleic acid of SARS-CoV-2 [8, 12, 13].

Health care professionals (HCPs) are at the front line of the COVID-19 outbreak response [14, 15]. As such, contact and droplet precautions should practice, and health care professionals must use personal protective equipment (PPE) [16–18].

Globally, Coronavirus disease-2019 disrupts health care systems. It is crucial to protect those most impacted by COVID-19, sustain gains made to address other infectious diseases, and maintain people’s access to life-saving health services [19]. The contained outbreak could significantly influence the global economy in the short run. The costs reduce by investing in public health systems in all countries. But, attention is needed for less developed countries that the health care system is weak [20]. The COVID-19 pandemic will create stress on the health care system in Africa [21, 22].

A qualitative study from China indicated that health care providers volunteered and tried their best to provide care for patients. They are challenged by working in a very new context, exhaustion due to heavy workloads and protective gear, the fear of becoming infected and infecting others, feeling powerless to handle patients’ conditions, and managing relationships in this stressful situation [23]. Several factors may influence the readiness of health workers to deliver essential services, including re-deployment of staff to treat increasing numbers of patients with COVID-19 and the absence of health workers in quarantine. The combination of increased workload and reduced numbers of health workers is likely to pose a severe strain on the capacity to maintain essential health care services [24].

Infection control interventions to reduce transmission of COVID-19 include universal source control, early identification and isolation of patients with suspected disease, the use of appropriate PPE when caring for patients with COVID-19, and environmental disinfection are obligatory in the health care settings [25]. The 74.7% of study participants believed that it was necessary to ask patients to sit far from each other, wear masks while in the waiting room, and wash hands before getting in the dental chair to decrease disease transmission, as shown in a study by Khader et al. [26]. Findings from Pakistan also stated that 13.8% of participants remove the mask while talking to the patient, 20.2% reused it, 44.9% correctly used the yellow-coded bag for disposal, 93.9% wear masks in clinics, and 94.6% wear masks on hospital premises [27]. Overcrowding, limited infection control material, less commitment of HCPs to the policies and procedures, insufficient training, and lack of policy and procedures of infection control practice identified as barriers [28].

Presently, studies are emerging regarding the COVID-19 pandemic in different aspects. However, a few have explored barriers that affecting the practice of preventive measures for the COVID-19 among health professionals. Therefore, there was a need to fill these research gaps in the study setting.

## Methods

### Study setting, design, and period

In this phenomenological study, key informants working in public health facilities of the Gamo zone, southern Ethiopia, were involved, from June 10–30, 2020. Gamo zone is one of the administrative zones in Ethiopia. It is bordered by Wolayta, Dawro, and Gofa zones in the North, Lake Abaya in the northeast, and Amaro special woreda and Dirashe special woreda in the southeast, and South Omo in the southwest. The administrative center of the Gamo zone is Arba Minch town. Gamo zone has one administrative town and 13 woredas. It hosted five hospitals (one general and four primary hospitals), 56 health centers, and 299 health posts.

### Sampling

A purposive sampling method was employed to recruit key informants. Chief executive officers, medical directors of the hospitals, health center heads, environmental health workers, chief nursing director, matron, heads in each ward or unit, and case team leaders involved for the key informants’ interview (KII). In this study, sixteen key informants were involved, based on idea saturation.

### Data collection method

Two interviewers were involved, and the information was collected using a semi-structured interview guide. The interview guide was developed based on expert opinion, knowledge, and skill of investigators and existing works of literature (Table 1) (Supplementary file 1). Interviewers trained, and key informants advertised in each participating health facility to involve in the study. The aim of the study explained for those who zealous to the participant. Then, to meet the study objective, pillar participants were purposively selected by the interviewer and involved in the KII. At the outset, the socio-demographic characteristics of each participant were documented after getting written and signed consent. Each session of the KIIs was recorded by an audiotape recorder, and keynotes took. The interview was conducted in a private room, which comfortable for the participants.

**Table 1:** Major points of the interview script

Topics in the interview script	
What do you think about COVID-19?	
How health professionals prevent themselves and patients from COVID-19?	
What do you think about precautionary measures?	
What are the perceived barriers to the practice of precautionary measures?	
What activities done in the health facility to prevent the spread of COVID-19?	
What do you suggest to improve the practice of health care providers to tackle the COVID-19 pandemic?	

### Trustworthiness

Trained and experienced interviewers collected the information. The interviewers communicated and discussed the points that they faced during the data collection period daily. The well-established, expert commented, reviewed semi-structured interview guide used to collect the data. The information was sought from participants with relevant expertise and experience, a neutral view of the investigator maintained, and participants probed and who had much on the issue selected to ensure trustworthiness. The participant’s variations were managed by involving KIs with different positions and responsibilities in the health facilities. Original audios and written transcripts re-checked to develop themes to achieve data credibility. NVivo 12 Pro software was used to enhance the maintenance of connections between participant descriptions and researcher synthesis. The result was presented alongside direct quotes from participants. The recorded interview transcripts, translations, and demographic summary of participants were maintained, and keynotes took during and after (during analysis) the interview to achieve the dependability of standards.

### Data analysis

The two investigators independently heard the audios of each interview that transcribed verbatim in the local language more times until they familiar with the participant’s information. Then, translated into English language transcripts by those investigators, compared, and argued for discrepancies. The investigators read and re-read the transcripts to familiarize themselves with the idea and developed memos and codes line by line. Then, refined and compared the emerged new themes and sub-themes. The investigators discussed until agreement regarding the inconsistencies, new ideas of the participants, emerged themes and sub-themes daily, and the data collection continued until idea saturation. To analyze that data, thematic content analysis was employed by using an inductive approach in NVivo 12 Pro software.

## Results

In this interview, sixteen-key informants were involved with mean age and standard deviation of 34.7 ± 8 years old. Of the key informants, 10 (62.5%) were male, and 13 (81.3%) had an educational level of BSc (1st degree). Out of participants, 13 (81.3%) participants served five or more years in the health facility. Eleven (68.8%) participants were health professional qualifications of public health, and four (25%) were nurses. Overall, six main themes and twenty-six sub-themes emerged in this study (Table 2).

**Table 2:** Main themes and sub-themes emerged in this study

Main themes	Sub-themes
Theme 1:Overview	Origin
	Causes
	Mode of transmission
	Sign and symptoms
	Treatment
	Preventive measures
Theme 2:Consequences	Stress on the HCPs
	The socio-cultural and economic crisis
Theme 3:Perceived practice of preventive measures	Using PPEs
	Handwashing and using alcohol and sanitizer
	Avoid contact
	Appropriate waste disposal
Theme 4: Perceived barriers	Community influence
	Health care provider related barriers
	Institutional barriers
	Lack of communication and support
Theme 5:Newfangled activities	Launching testing center and other services
	Controlling patients, visitors, and staffs
	Taskforce organization and surveillance
Theme 6: Suggestions for improvement by HCPs	Conduct outreach services and involve community leaders
	Control transportation
	Strengthen multidisciplinary teamwork
	Motivation and capacity building for staffs
	Fulfill necessary supplies
	Monitor activities continuously
	Mass screening and campaign

### Overview of COVID-19

The majority of participants stated that the origin of COVID-19 was from the markets of Wuhan province, China, after eating animal meat (bat). It is caused by a virus and the disease named by year of occurrence, called Coronavirus disease-2019 (COVID-19).

*“Coronavirus disease-2019 (COVID-19) originated from Wuhan province, China after eating bat meat”* (Participant 10).

*“The name of disease given by the virus name and year of occurrence, which called Coronavirus Disease-2019”* (Participant 14).

The most commonly reported modes of transmission of the COVID-19 were physical contact, air and droplets, and body fluids. Dry cough, fever, shortness of breathing, sore throat, back pain, and sneezing were symptoms reported by the study participants.

*“The mode of transmissions are physical contact, air, droplets, handshaking, and different body fluids”* (Participant 15).

*“The sign and symptoms of COVID-19 are fever, the difficulty of breathing/shortness of breathing, and sore throat”* (Participant 16).

*“The sign and symptoms of COVID-19 are fever, back pain, sneezing, and dry cough”* (Participant 12).

The majority of the participants responded that homestay (social distancing), physical distancing, avoiding public gathering, hand washing, applying facemask, and using hand sanitizer or alcohol could prevent the transmission of the COVID-19. There is no proven drug for the COVID-19 until now. Nevertheless, some participants stated high dose antibiotics used as treatment and taking cooked hot foods and fluids recommended as supportive management.

*“There is no vaccine and treatment for Coronavirus Disease-2019. But, the best prevention way is homestay; others are not got to a public gathering, physical distancing, washing hands with soap and water, using alcohol or sanitizer, and using facemask at market or place where so many individuals gathered”. Besides, to above-stated prevention ways, social or physical distancing at least 2 m or 6 ft. Reducing transport, not got to “Lekiso” or burial places, “Ikub” and Edir” places”* (Participant 4).

*“The prevention ways are social distancing, frequent washing with water and soap by rubbing hands for 20 seconds, using alcohol and sanitizer in areas where water is not available. Two staffs from our sectors trained on COVID-19 prevention and treatment, and they stated that we could treat COVID-19 by antibiotics by increasing the dose (at least by Amoxicillin) and by taking cooked hot food and fluids”* (Participant 3).

### Consequences of COVID-19 pandemic

Tension on health professionals and absence from work, socio-cultural, and economic crisis were the main consequences of the COVID-19 pandemic. Currently, it is the utmost public health issue all over the world.

*“COVID-19 is the most severe and resulted in tension on HCPs and economic crisis. It is possible to control the transmission but challenged with the societal culture and the way of living”* (Participant 7).

### The perceived practice of preventive measures of the COVID-19 pandemic in health professionals

The majority of the health professionals prevented themselves and others who came to the health facility by using personal protective equipment (PPEs) (facemask, glove, and gown), frequently washing hands with soap and water. They continuously maintained recommended physical distance as needed, distance beds in the ward, controlling the number of visitors, and order the visitors to use a facemask. All most all the health professionals use hand sanitizer or alcohol after any contact with patients. Patients with cough and high fever identified and isolated start from pre-triage. The other infection prevention practices (IPPs) are also applied in the health facility, such as an appropriate waste disposal system.

*“Health care professional prevent themselves and patients or any person who visits the health facility by using different kinds of masks (surgical mask, N-95 facemask, and locally produced masks), frequently washing hands with soap and water, and by using hand sanitizers”* (Participant 1).

*“Health care workers, preventing themselves and others by using a facemask, which produced locally, and some bought their N-95 facemask and hand sanitizer. They wash hands with soap and water in the main get and emergency room only due to a shortage of handwashing facilities. The entrance of patients and visitors controlled in the main get. One person or supporter for severely ill patients and only patient if stand-alone can get into the facility. All individuals who enter the facility must apply a facemask and wash hand in the main get”* (Participant 8).

*“In the case of the pediatric ward, the patient came after passing pre-triage, and all the staff practice standard precautionary measures such as using a facemask, donning gloves, frequently washing hands, and use sanitizers. If the patients with cough came to the ward, there is isolation room until diagnosis and if we suspected COVID-19 sample sent to the testing center”* (Participant 11).

*“All health professionals have a facemask and maintain physical distancing. However, it is difficult in case of maternal, child health services, family planning, delivery, and postnatal care because there is direct physical contact”* (Participant 7).

*“The health care workers preventing the pandemic by infection prevention practice such as avoiding or stored contaminated materials in specified places and follow the adequate waste disposal mechanisms, using PPEs (facemask, gloves, and gowns)”* (Participant 15).

### Perceived barriers for the practice of preventive measures for COVID-19

#### Community influence

The main factor that affects the health care providers’ practice of preventive measures for the COVID-19 pandemic were lack of awareness of the community, negligence, and ignorance, not using a facemask and using incorrectly, and miss conception about the disease.

*“There is ignorance and negligence in the community to accept the practice of prevention ways or to avoid the previous culture while came to our facility”* (Participant 13).

*“Most community members who came to institution perceived that there are no any COVID-19 and it is false and politics, not a disease. It is a common cold, which is cured easily. Because no one is infected and no one is dead in our surrounding”* (Participant 3).

*“There is a lack of awareness in the community who came to the institution even if awareness created by different stakeholders by using montarbo and microphones. They came without a facemask, and some apply facemask inappropriately”* (Participant 7).

#### Health care provider related barriers

The national response on COVID-19 prevention in a government organization allowed pregnant and breastfeeding staff, advanced age staff, and staff with chronic diseases to stay in the home. However, in contrast to these, health care providers with those stated issues are obligated to do work in the health care institutions. Therefore, this may affect the willingness and motivation of the staff.

*“The main challenge in this facility to practice precautionary measures in line with standards and emergency operation is that pregnant and breastfeeding staffs, aged staffs, and staffs with chronic diseases like asthma and diabetes mellitus were obligated to work and not allowed to stay at home”* (Participant 3).

As reported by the utmost participants, negligence and ignorance were the most speculated factor or challenges in the practice of preventive measures for the COVID-19 pandemic.

*“Regarding the practice precautionary measures to prevent COVID-19, most staffs practice the measures even if there are some negligent and ignorant staffs due to no occurrence of the case around the town”* (Participant 16).

#### Institutional barriers

The shortage of PPEs (facemask and glove), scarcity of hand cleaning solutions (alcohol and sanitizer), and inadequate training and trained staffs were the most common barriers that influence the practice of preventive measures. Similarly, unavailability of guidelines, water shortage, lack of duty, OPD, and emergency rooms were also point outed by key informants.

*“The barriers are the absence of hand sanitizer, shortage of alcohol, facemasks, and other PPE since our district is new, and the majority of the thinks are limited. There is no screening in our institution as well as in our district even with infrared thermometer”* (Participant 5).

*“The barriers that affect the practice of precautionary measures in our institution are … , shortage of supplies (facemask, gloves, etc.), inadequate OPD, and emergency rooms to maintain the recommended physical distance. The other factors are..., unavailability of guidelines to orient the staffs”* (Participant 15).

*“There is no training and psychological support for HCP to avoid stress and to stabilize the situation. The budget issue is the main concern to fulfill the necessary materials and training all the health care providers. As our health facility, there is no water, we buy 20 litter/ 10 birr per day water from surrounding, and we use rainwater, which strode in the tanker, there is also a shortage of alcohol and sanitizer, and district health office gave two litter alcohol and sanitizer”* (Participant 4).

*“The barriers that affect the practice of precautionary measures in our institution are inadequate duty rooms for staffs that many staffs exchangeable used a single room for a period and if one individual from those infected the risk contamination is most likely. The other challenge is the shortage of hand sanitizer and facemask. The recommended duration of use of single facemask is 8 hour, but most staffs use more than a week”* (Participant 6).

*“The main problem in this health facility is that as different teams or stakeholders, commented there is no triage. However, we simply isolated patients with cough and gave priority for those individuals”* (Participant 3).

#### Lack of communication and support

Some of the study participants highlighted that lack of communication, support from the community and government, and lack of attention and recognition for staff were factors that influence the practice of precautionary measures for the COVID-19 pandemic in the health care facility.

*“The barriers that affect the practice of precautionary measures are attention and recognition not given for health care professionals …*. T*here is no support from the town health office, zonal health department, and task force in the zone rather than taking the suspected or confirmed cases to the treatment center. Staffs in other government organization apply standard and recommended facemask, but we struggled to serve the community by locally produced and below standard facemasks”* (Participant 8).

*“The other factors are lack of communication of our institution with supporting organization or universities to train the staffs and unavailability of guidelines to orient the staffs”* (Participant 15).

#### Newfangled activities in the health facility after the occurrence of the pandemic

Organization of fever clinic and pre-triage service, hand washing station, and screening with infrared thermometer or thermo-scan in the get were the major newfangled activities after the occurrence of the COVID-19 pandemic. Individuals are also controlled and obligated to apply facemask while entering the health facility.

*“Activities that conducted after the occurrence of the pandemic are an organization of fever clinic that provided by trained staffs with an infrared thermometer, pre-triage service, and COVID-19 testing center launched. Handwashing facilities organized in a different place and all persons are obligated to apply facemask”* (Participant 12).

*“The activities conducted in the facility after the occurrence of the pandemic were a team (taskforce) created and a team of health professionals trained in the zonal level. There is integration with health extension workers at the health post, and those health extension workers conducted surveillance at least 20 households per day to identify the suspected cases”* (Participant 3).

*“The activities conducted in our facility after the pandemic was the construction of new handwashing station, screening in the get, distancing patient bed in the ward, reducing the number participates in meeting”* (Participant 2).

#### Suggestions by health professionals for the improvement

Involving and integrating the awareness creation activities with the community leaders was very important. Strengthening and monitoring the activities of different task forces, extensive training for health professionals and leaders in the health facility also suggested by the study participants. Surveillance with mass screening, outreach services to create awareness in the community, transportation control, and attention and recognition for health professionals were the other concerns, which recommended.

*“ … the community leader must intervene on this to save the community from this pandemic. The community practicing already an existing culture in “Eder” “Ekub“ and “Lekiso“ places, that the task force, the town health office, and the zonal health department must support the health professionals or team to create awareness in the community … “* (Participant 8).

“*… awareness creation must continue in the community … transport access must arrange … for the staffs who came far from the health facility”* (Participant 7).

*“ … task force activities are key roles to prevent the spread of infection in the community. Therefore, those activities must strengthen and frequently monitored rather than one session or one-week activity”* (Participant 1).

*“ … the leaders in the facility must train on COVID-19 prevention aspects before anyone that the leaders are the main responsible person or individual to orient or to facilitate the health professionals or staffs to practice prevention measures”* (Participant 2).

*“ … mass screening needed to identify the case, staffs and all the community must apply a facemask and use hand sanitizer … “* (Participant 12).

## Discussion

### Summary

The novel coronavirus is a global medical pandemic that resulted in stress on the health care system. There is no proven drug for this disease. Because of the high contagiousness, strictly practicing precautionary measures can save the lives of many populations. Studies are emerging related to the epidemiological aspects, social, political, and economic consequences of the COVID-19. Nevertheless, few are exploring barriers. As such, this aimed to fill this research gap in Ethiopia. The study found that negligence and ignorance, shortage of PPEs, lack of infrastructure, lack of training, lack of communication, and lack of attention and recognition for staff were the most common barriers to the practice of precautionary measures for COVID-19 in the health facility.

### Comparison and contrast with exiting evidence

Less commitment of health care workers to the policies and procedures was the main factor that the health professionals did not practice prevention ways and affected with the COVID-19 [28, 29]. This study also reconnoitered that negligence and ignorance among health care workers were the most speculated factor or challenges in the practice of preventive measures for the COVID-19 pandemic. The reason is that most people, including health professionals, have miss conception about the disease and the contagiousness, and due to that case, they do not give attention to the prevention practices and abandon due to not the occurrence of the disease in the surrounding that they reside.

This study found that a shortage of PPEs (facemasks and gloves) and scarcity of hand cleaning solutions (alcohol and sanitizer) had the main barriers. These findings also are shown in studies conducted elsewhere [28–35]. These are the fact that health professionals are obligated to do the procedures or help patients with available resources (PPEs), and a breach in standard precautions inevitable. These resulted in the health professionals more vulnerable to the infection and more likely to transmit the disease (COVID-19) to others. Studies conducted in Ethiopia [32], Pakistan [28], and China [31] reported that lack of access to infection prevention training or insufficient/inadequate training in infection control measures resulted in the poor practice of precautionary measures for the COVID-19. A result of systematic review also supplements the findings of the above-stated literature. Health professionals were not trained on the infection and how to use PPEs, and they reported it was a problem when training was not mandatory [34]. The other study stated institutional (training and education, policies, and management expectations) factors are essential to improve preventive measure practices [35]. In line with this, this study found that a shortage of training for the staff (health professionals) had one of the barriers that affect the practice of precautionary measures for COVID-19. Provide training for the health professionals on overall aspects of the COVID-19, including precautionary measures in the health care facility, can improve preventive measures practices.

Evidence showed that tied to the guideline itself and how it communicated, support from managers, workplace culture, lack of physical space, and poor design of the hospitals affecting HCP’s ability and willingness to follow IPC guidelines. Besides, it affects infection prevention practice when managing respiratory infectious diseases [32, 34, 36]. This study also identified that lack of communication and support, unavailability of the guidelines, shortage of water supply, inadequate duty, OPD, and emergency rooms were barriers to the practice of preventive measures. The reason is that updated guidelines, communication with the different supporting teams and support from concerned bodies, adequate facilities to provide quality services with the standard are pivotal to practice precautionary measures for COVID-19 in the health facility.

Lack of attention and recognition for staffs, lack of motivation and they obligated to do work, and lack of awareness, negligence, and ignorance of the community while coming to health care institution were also pointed out in this study as barriers that practice of preventive measures for COVID-19 among health professionals. The other barriers also identified, such as not using a facemask and using incorrectly, and miss conception about the disease. These are facts that give attention and recognition for those in the front line, the motivation by fulfilling the thing that urgently needs as much as possible, and community vigilance can improve the practice.

### Limitation

Only two interviewers were involved to ensure consistency and bracketing. However, those interviewers were experts on qualitative data collection and had full information and idea about the study protocol. The ethical review board not allowed other qualitative data collection methods rather than KIIs. These were due to the nature of the disease transmission and the state emergency declaration. The sample size was relatively small, and only key informants were involved in this study. These may affect the transferability of the study results.

### Implications

This study can be input for the policymakers and program evaluators to design appropriate strategies and planning. These initiate training and capacity building in the health institutions based on the identified gaps. Health care providers and other stakeholders benefited from this study. The finding serves as a tool for public policy and case management teams to evaluate the prevention and control measures for novel coronavirus in the health care institution. It also provides the basic framework for future studies in assessing and comparing the performance of interventions.

## Conclusions

This study found a gap in practicing preventive measures for COVID-19 in health facilities among health professionals due to multiple barriers. Shortage of PPEs and hand cleaning solution, shortage of training, negligence, and ignorance of HCPs, and poor infrastructure in the facility were the most common barriers that need immediate attention. Lack of motivation and obligation to do work and lack of recognition for HCPs were also identified as barriers to practice preventive measures for COVID-19. Therefore, the government, the ministry of health, non-governmental organizations, and other voluntary supports should fulfill the necessary supplies in the health facilities and improve the infrastructures. Different capacity-building (individual, organizational and systemic) activities needed such as, training and workshops, mentorships, seeking volunteers with expertise, and raising public awareness. Besides, attention and recognition must give to the health care professionals.

## Supplementary Information

**Additional file 1: Supplementary file 1**. English version interview guide.

## Data Availability

The datasets (audios and transcripts) generated and/or analyzed during the current study are not publicly available due to anonymity issue but are available from the corresponding author on reasonable request.

## Abbreviations

COVID-19: Coronavirus Diseases-2019
HCPs: Health Care Professionals
HCWs: Health Care Worker’s
KII: Key Informant Interview
OPD: Outpatient Department
PPEs: Personal Protective Equipment’s
