# Supplementary material for: Perceived barriers to the practice of preventive measures for COVID-19 pandemic among health professionals in public health facilities of the Gamo zone, southern Ethiopia: a phenomenological study
Source: BMC Public Health. 2021 Jan 22;21:199. doi: 10.1186/s12889-021-10256-3 (PMC7820827; doi:10.1186/s12889-021-10256-3)
Supplement: Supplementary file 1 — Additional file 1: Supplementary file 1. English version interview guide. [file 12889_2021_10256_MOESM1_ESM.pdf]

Interview Guide

A. Key informants background information

1. Key informant code:________
2. Age:_______
3. Sex: ______
4. Educational level: __________
5. Work experience (in year):_________
6. Job category__________
7. Position/status:______

B. Interview script

1. What do you think about COVID-19? (Origin, cause, mode of transmission, preventive measures, and treatment)
2. How health care professional prevent themselves and patients from COVID-19 transmission?
3. What do you think about precautionary measures for COVID-19 pandemic in the health facility?
4. How the staffs practicing the precautionary measures for COVID-19 pandemic in the health facility?
5. What are the perceived barriers for the practice of precautionary measures for COVID-19 pandemic by health care professionals?
6. What activities done in the health facility to prevent the spread of COVID-19?
7. What you suggest to improve the practice of health care providers to tackle COVID-19 pandemic?
8. Any additional points, you may state related to COVID-19, and barriers.

I completed the interview, thanks a lot!
